# HOME CARE AIDE-LED RESISTANCE EXERCISE FOR MEDICAID HOME AND COMMUNITY-BASED SERVICES CLIENTS

**DOI:** 10.1093/geroni/igz038.2929

**Published:** 2019-11-08

**Authors:** Margaret Danilovich, Laura Diaz, Jody Ciolino, William Healey, Gail Huber, Daniel Corcos

**Affiliations:** Northwestern University, Chicago, Illinois, United States

## Abstract

Home Care Aides (HCAs) assistance is the most common care provision to help clients with activities of daily living, but exercise is not currently provided, despite well-established health benefits. We developed a resistance exercise intervention using a stakeholder panel of physical therapists, HCAs, and clients. Stakeholders suggested a mobile application that played exercise videos. We then enrolled 128 HCA-client dyads (93% african american & 79% female) and randomized home care aides to lead the intervention with their clients in addition to usual care for 6 months or continue usual care. Our aims were to 1) evaluate the feasibility of the intervention and 2) determine the effects of the intervention on client physical performance, frailty classification, self-reported health, and strength. 96% of HCAs reported the training session successfully prepared them to lead exercise and we had no adverse events. There was high attrition rates among dyads (28.13%) at 6 months which was worse among HCAs (55%). We found no statistically significant differences between study arms. However, the range of completed sessions was 1-34 out of 52 possible sessions. Program evaluation indicated high satisfaction among clients and HCAs (8.9 & 8.6/10). HCA attrition limited client participation, technology did not facilitate exercise completion, and client motivation was a barrier to adherence. Results show it is feasible to train HCAs to lead exercise, however, given high turnover in the HCA profession, alternative implementation models should be studied. While the app use was an innovative way to engage older adults in exercise, technology challenged HCAs.

